# Catch-up Growth at Term Equivalence in Extremely Premature Small for Gestational Age Infants Compared with Extremely Premature Appropriate for Gestational Age Infants

**DOI:** 10.4274/jcrpe.galenos.2019.2019.0044

**Published:** 2019-11-22

**Authors:** Hüseyin Anıl Korkmaz

**Affiliations:** 1 Manisa City Hospital, Clinic of Pediatric Endocrinology, Manisa, Turkey

**Keywords:** Nutritional thrift, small gestational age, postnatal weight gain

## Dear Editor,

Extremely premature small for gestational age (SGA) children are more prone to medical conditions such as insulin resistance, type 2 diabetes mellitus, precocious puberty, polycystic ovarian syndrome, hypertension, hyperlipidemia and cardiovascular disease (1,2,3,4). There is a balance between prenatal and postnatal weight gain in life. This balance allows the safe storage of fat in the subcutaneous adipose tissue. SGA children have a greater risk of endocrine and metabolic problems if there is mismatch between prenatal and postnatal weight gain (1,2,3,4).

SGA fetuses need to make a metabolic organization for surviving, if they do not have an adequate supply from the placenta and these fetuses tend to economize their resources. Thus, these fetuses send a blood supply to their brain for maintaining their life, while their bodies receive an inadequate blood supply. Their organs (pancreas, liver, kidneys) also receive an inadequate blood supply in the prenatal period (1,2,3). Pancreatic beta cells can not tolerate more energy intake in later life if there is mismatch between prenatal and postnatal weight gain and decreased insulin sensitivity may occur (1,2,3,4). This mismatch is also associated with central adiposity in later life. These infants are also susceptible to precocious puberty, polycystic ovarian syndrome, hypertension, hyperlipidemia. They tend to have a lower risk for insulin resistance and cardiovascular disease, as long as they receive a restricted food supply in later life as in their prenatal period (1,2,3,4). Ng et al (5) reported that extremely premature SGA infants achieved catch up growth with postnatal nutrition, but they tend to have a greater risk of insulin resistance, type 2 diabetes, polycystic ovarian syndrome, hypertension, hyperlipidemia and coronary artery disease because of nutritional thrift.

Catch-up growth is important for reaching higher adult height in extremely premature SGA infants, but nutritional thrift should be considered for prevention of insulin resistance, type 2 diabetes mellitus, polycystic ovarian syndrome, hypertension, hyperlipidemia and cardiovascular disease. Mismatch between prenatal and postnatal weight gain may cause more serious medical disorders than short stature. Nutritional balance should be provided for mitigating the risk of metabolic and endocrine disorders.
